# Potential Modulation of Sirtuins by Oxidative Stress

**DOI:** 10.1155/2016/9831825

**Published:** 2015-12-14

**Authors:** Leonardo Santos, Carlos Escande, Ana Denicola

**Affiliations:** ^1^Laboratorio de Fisicoquímica Biológica, Instituto de Química Biológica, Facultad de Ciencias, Universidad de la República, Iguá 4225, 11400 Montevideo, Uruguay; ^2^Laboratorio de Patologías del Metabolismo y Envejecimiento, Institut Pasteur de Montevideo, Mataojo 2020, 11400 Montevideo, Uruguay

## Abstract

Sirtuins are a conserved family of NAD-dependent protein deacylases. Initially proposed as histone deacetylases, it is now known that they act on a variety of proteins including transcription factors and metabolic enzymes, having a key role in the regulation of cellular homeostasis. Seven isoforms are identified in mammals (SIRT1–7), all of them sharing a conserved catalytic core and showing differential subcellular localization and activities. Oxidative stress can affect the activity of sirtuins at different levels: expression, posttranslational modifications, protein-protein interactions, and NAD levels. Mild oxidative stress induces the expression of sirtuins as a compensatory mechanism, while harsh or prolonged oxidant conditions result in dysfunctional modified sirtuins more prone to degradation by the proteasome. Oxidative posttranslational modifications have been identified *in vitro* and *in vivo*, in particular cysteine oxidation and tyrosine nitration. In addition, oxidative stress can alter the interaction with other proteins, like SIRT1 with its protein inhibitor DBC1 resulting in a net increase of deacetylase activity. In the same way, manipulation of cellular NAD levels by pharmacological inhibition of other NAD-consuming enzymes results in activation of SIRT1 and protection against obesity-related pathologies. Nevertheless, further research is needed to establish the molecular mechanisms of redox regulation of sirtuins to further design adequate pharmacological interventions.

## 1. Introduction

Sirtuins are a conserved family of enzymes, originally defined as histone deacetylases (class III HDAC) [1]. They deacetylate not only histones but also other proteins. In addition, they catalyze the hydrolysis of lysines modified with longer acyl chains (deacylase activity) [2]. Unlike classes I, II, and IV HDAC that utilize zinc for catalysis, sirtuins use a complex mechanism depending on cofactor NAD that already discloses a fine-regulated activity.

Since the discovery of yeast Sir2 (Silent Information Regulator 2) 30 years ago [3], the founding member of the family, an intensive research went on to elucidate the biological functions of sirtuins, especially after the early found connection of sirtuins with lifespan [1, 4]. The number of publications grew exponentially in the search of potential activators or inhibitors of sirtuins that fight against metabolic disorders, cancer, and even aging [5].

In* S. cerevisiae*, besides Sir2, four more sirtuins were described, Hst1–4. In* C. elegans*, four homologs of yeast Sir2 were named Sir2.1–2.4, whereas seven paralogs were described in mammals, SIRT1–7. Phylogenetic analysis groups the mammalian SIRT1, SIRT2, and SIRT3 as subclass I which shows close homology to yeast Sir2, SIRT4, and SIRT5 as subclasses II and III, respectively, and SIRT6-SIRT7 in subclass IV [6].

The seven mammalian SIRT differ in sequence (although they all share a conserved catalytic core), in subcellular location, enzyme activity, and substrate specificity. The list depicted in Table 1 is by no means comprehensive since new* in vivo* substrates and specificities are discovered every day. The most studied human isoform is SIRT1, a nuclear protein reported to regulate critical physiological processes and associated with chronic inflammatory diseases and metabolic dysfunctions like diabetes, obesity, aging, and even cancer [7].

This review focuses on the effect of oxidative stress on structure and activity of sirtuins and the biological consequences of their redox regulation. Understanding the role and mechanism of action of sirtuins in the context of a pathophysiological inflammatory condition will help to identify novel interventions to manage important chronic diseases.

## 2. Sirtuins Structure

**Figure 1:**
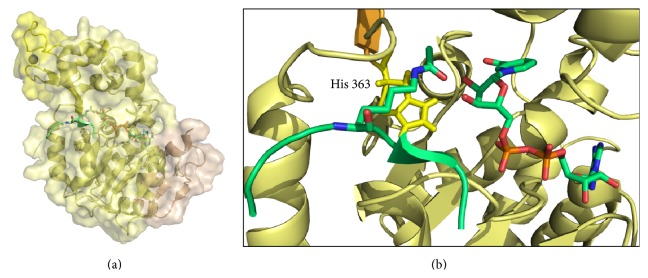
Structure of sirtuins. (a) Crystal structure of a partial sequence of hSIRT1 (PDB 4KXQ) with bound substrates, acetylated peptide, and NAD. The catalytic core is depicted in yellow with the Zn^2+^ binding domain. (b) Zoom of catalytic site with the catalytic histidine colored in yellow.

Crystal structures of sirtuins from archaea to eukaryotes show a central catalytic core comprised of 245 residues. The core is made up of a large domain containing a Rossmann fold typical of NAD-dependent proteins and a small domain containing a Zn^2+^ ribbon motif, separated by a cleft where the peptide substrate binds (Figure 1). The NAD molecule adopts an extended conformation binding to a grove between the two domains with the adenine base facing the large domain and the nicotinamide group close to the small domain (Figure 1). SIRT1 is the biggest isoform with extended N- and C-terminals very flexible, unstructured, which offers more sites of activity modulation (posttranslational modifications, interaction with proteins and ligands).

The Zn^2+^ binding site is composed of three antiparallel beta strands containing two Cys-X-X-Cys conserved motifs separated by 15–20 residues that coordinate a single zinc ion that has an important structural role. It has long been known that mutation of these cysteine residues by alanine causes loss of activity [8]. Although the zinc tetrathiolate is fairly exposed, only high concentrations of zinc chelator were able to disrupt it with the corresponding loss of activity [9]. Another report on* P. falciparum* Sir2 obtained the inactive apoenzyme by treatment with potent zinc chelator and restored activity upon reconstitution with exogenous zinc chloride [10].

The zinc ion is located in the small domain, far away from the NAD binding pocket, excluding the possibility of participation in the catalysis, in contrast with other HDAC types where zinc is part of the catalytic mechanism [11].

## 3. Enzymatic Activities of Sirtuins

**Figure 2:**
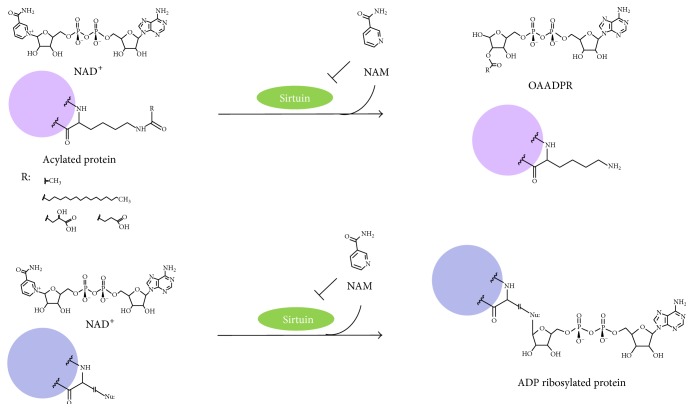
Scheme of reactions catalyzed by sirtuins. Deacetylation is the most common reaction catalyzed by sirtuins, but some sirtuins catalyze deacylation of other posttranslational lysine modifications and mono ADP ribosylation. NAM = nicotinamide, OAADPR =* O*-acetyl-ADP-ribose.

Sirtuins are defined as protein deacylases. They catalyze the reaction depicted in Figure 2 using NAD as a cofactor, yielding the deacylated protein, nicotinamide (that displays inhibition by product), and acylated ADPR as final products.

Kinetic studies and isotope exchange indicate that sirtuins first bind the acetylated substrate, followed by NAD binding to form a ternary enzyme complex where the carbonyl oxygen of the acetyl group attacks ribose C1′ to form O-alkylamidate intermediate. Crystal structures of binary complexes were solved between Sir2-like enzyme and NAD [9], or ADP-ribose [12], or acetylated p53 [13]. Moreover, the crystal structure of a ternary complex was reported between yeast Hst2, an acetylated histone peptide, and a nonhydrolyzable NAD analog [14]. Crystal data confirm the peptide substrate binds in a narrow channel that positions the acylated lysine residue near the nicotinamide ring of NAD (Figure 1). Upon peptide binding, a conformational change on the NAD site must occur to facilitate the nucleophilic attack on ribose C1′ to cleave the nicotinamide-ribosyl bond, first step in the catalytic pathway. A conserved histidine residue (H363 in hSIRT1) has been identified as critical for the catalysis, first acting as a general base hydrogen bonded to 3′′ OH-ribose and, then, as a general acid protonating the lysine residue in the last step of the catalysis.

Besides protein deacetylation, it was early recognized that sirtuins can also catalyze ADP ribosylation of a protein acceptor (or the enzyme itself) via a similar mechanism (Figure 2) [14–17].

More recently, it has been found that some sirtuin isoforms previously considered poor deacetylases are actually good deacylases; that is, they catalyze the hydrolysis of lysine amides derivatized with a longer-chain carboxylic acid, for example, succinate or malonate. Indeed, SIRT5 functions as desuccinylase or demalonylase [18], whereas SIRT6 functions as demyristoylase [2, 19]. Moreover, SIRT6 deacetylase activity has been recently shown to be regulated by free-fatty acids* in vitro*, opening the possibility that fatty acids might be acting as endogenous regulators of sirtuin activity* in vivo* [2].

Acetylation is an important posttranslational modification even outside chromatin. The acetylome shows that many proteins are acetylated as a mechanism of regulation of cellular function, and it is even possible that is as common in cellular life as phosphorylation [20, 21]. Comparative studies on* Drosophila* and humans have demonstrated that acetylated lysines are highly conserved [22, 23]. An acetylome peptide microarray has been described that reveals new deacetylation substrate candidates for all sirtuin isoforms [24].

## 4. Sirtuins and Oxidative Stress

As mentioned above, increasing evidence supports the role of sirtuins in the regulation of cellular homeostasis, in particular metabolism and inflammation [25, 26]. During conditions of metabolic stress, like obesity and metabolic syndrome, an oxidative stress environment is created, mainly due to a state of chronic inflammation. Based on the key role of sirtuins in the regulation of metabolic responses [27, 28], it is pertinent to ask how changes in the redox status of the cells affect the activity of sirtuins and what are the biological consequences of these alterations.

Oxidative stress, considered as an overwhelmed generation of reactive species (ROS/RNS) or a general disruption of redox cellular homeostasis [29, 30], can affect the activity of sirtuins at different levels:

1. Inducing or repressing the expression of SIRT gene.
2. Posttranslational oxidative modifications of SIRT.
3. Altering SIRT-protein interactions.
4. Changing NAD levels.

### 4.1. Changes in Sirtuin Expression during Oxidative Stress

It has been observed that* mild oxidative stress conditions induce the expression of SIRT1*, changing its activity and thus affecting SIRT1 targets that are involved in the response to changes in the redox state of the cell [31–33]. The first major SIRT1 substrate identified was p53, a transcription factor involved in activating antioxidant genes like SOD2 (superoxide dismutase 2, MnSOD) and GPx1 (glutathione peroxidase) [34]. Another redox transcription factor deacetylated by SIRT1 (as well as SIRT2 and SIRT3) is FOXO3a which induces an antioxidant response via SOD2 and catalase expression [35–40]. PGC1*α*, a known substrate of SIRT1, is reported to regulate expression of mitochondrial antioxidants like SOD2 [41–43]. SIRT1 can deacetylate p65 NF*κ*B subunit diminishing its activity and, thus, the production of proinflammatory cytokines [44–46]. In addition, upon increased production of ROS at the mitochondria, induction of SIRT3 was observed [47]. It was reported that SIRT3 deacetylates and thus activates SOD2 reducing oxidative stress in the mitochondria [48]. In adult mouse hearts, SIRT1 was significantly upregulated (4-fold) in response to oxidative stress (paraquat injection) and, similarly, 3-fold increase in SIRT1 levels was observed in old versus young monkey hearts [49]. In the same way, modest overexpression of SIRT1 retarded age-dependent changes in the heart of transgenic mice [49]. Low levels of H_2_O_2_ promoted deacetylation of the tumor suppressor protein PLM in HeLa cells via SIRT1 and SIRT5 [50].

On the contrary,* exposure to high levels of H*_*2*_ *O*_*2*_ *or harsh oxidative stress resulted in* increased proteasomal degradation of SIRT1, desumoylation, and* enzyme inactivation* that leads to apoptosis [51]. Human monocytes exposed to high dose of H_2_O_2_ (250 *μ*M, 24 h) resulted in a significant decrease in SIRT1 activity (measured as levels of acetylated p53) and lower SIRT1 gene and protein expression [52]. Human lung epithelial cells exposed to oxidants (H_2_O_2_, aldehyde-acrolein, and cigarette smoke extract) presented decreased levels of SIRT1 concomitant with decreased SIRT1 activity [53]. A recent work on human endothelial cells showed no effect of low doses of H_2_O_2_ but a drastic drop to 50% SIRT1 activity after exposure to 100 *μ*M H_2_O_2_ for 30 min, along with a decrease in free thiol content of SIRT1 [54].

An interesting view suggested by Tong et al. [55] is that active sirtuins provide an adequate level of *O*-acetyl-ADP-ribose (OAADPR) (product of the reaction catalyzed by sirtuins with deacetylase activity, Figure 2) that readily converts to ADP-ribose and both may function as cellular signals. Increased ADPR/OAADPR levels protect cells from oxidative stress via two mechanisms: (1) inhibition of Complex I of the mitochondrial electron transport chain with concomitant lower production of ROS and (2) inhibition of glyceraldehyde-3-phosphate dehydrogenase, central enzyme in glycolysis, diverting glucose to the pentose phosphate pathway with the concomitant increase in NADPH, main reductant for detoxifying ROS enzymes.

### 4.2. Posttranslational Modifications (PTM) of Sirtuins

Phosphorylation was the first PTM found in SIRT1. SIRT1 is the most studied mammalian isoform although a crystal structure of the whole protein is not available and we rely on a simulation model [56]. Apart from the central catalytic structured core, SIRT1 has long C- and N-terminal domains which are flexible and disordered, not present in the other SIRT structures, and considered potential sites of enzyme regulation. Early mass spectrometry (MS) analysis detected several serine/threonine phosphorylation sites at the N- and C-terminal domains of SIRT1 [57]. Several kinases are known to phosphorylate SIRT1, and many of them are regulated by oxidative stress. CdkI (also known as Cdc2), a kinase involved in cell cycle progression and regulated by oxidative stress [58], phosphorylates SIRT1 in its C-terminus domain (T530 and S540) [57]. Mutations of these two sites on SIRT1 affect cell cycle progression [58]. SIRT1 is also phosphorylated by Casein Kinase II (CKII) in serines S154, S649, S651, and S683 [59]. CKII activity is tightly regulated by oxidative stress [60], and, indeed, ionizing radiation activates CKII, leading to SIRT1 phosphorylation and activation [59]. Phosphorylation of SIRT1 in different residues by AMPK has also been shown to regulate its activity mainly by affecting binding to its protein inhibitor DBC1 [61, 62]. AMPK is a key sensor and regulator of redox state of the cell and its biological activity is regulated by oxidative stress [63], although no direct link between oxidative stress and SIRT1 involving AMPK has been shown until now. Finally, phosphorylation of SIRT1 at different C-term residues has been shown to change its enzymatic activity. SIRT1 phosphorylation (T530) triggers a conformational change that increases its deacetylase activity [64–66]. Also, PKA-dependent phosphorylation of SIRT1 (S434) stimulates its activity [67]. Sumoylation at the C-terminal domain of SIRT1 (K734) has been detected and shown to increase activity as well [51]. Phosphorylation sites at the C-terminal of SIRT2 (S368, S372) were also reported to regulate enzyme function [68, 69]. In the case of SIRT6, phosphorylation at T294 and S303 were identified in a proteomic analysis, with no report on functional consequences [70, 71]. Another report shows that phosphorylation of SIRT6 at S338 by AKT leads to its degradation in breast cancer cells [72]. Moreover, mutation of that phosphorylation site made breast cancer cells more sensitive to chemotherapeutic agents [72].

Oxidative modifications of sirtuins are less well studied. Treatment of recombinant hSIRT1 with nitrosoglutathione (GSNO) was first reported [73] to modify C67 (located in the noncatalytic C-terminal domain) by S-glutathionylation, with no effect on basal deacetylase activity but loss of stimulation by resveratrol* in vitro* (although it has to be mentioned that the activity was measured using the fluorimetric assay that it is known to yield an artefactual activation of SIRT by resveratrol [74]). In this work [73], differential alkylation revealed 5 out of the 19 cysteines on human SIRT1 as reactive towards GSNO. Three of those five modified cysteines are solvent exposed residues (C67, C268, and C623) as indicated in the computer generated model of human SIRT1 structure [56]. However, in that same year 2010, it was published that treatment of SIRT1 with GSNO resulted in nitrosylation (not glutathionylation) of the enzyme with loss of deacetylase activity [75]. The residues modified (C387 and C390 from the mouse ortholog that coordinates the zinc ion) were different from those proposed previously [75]. These authors reported that treatment of intact HEK293 cells with GSNO resulted in nitrosylation of SIRT1 (SIRT1-SNO) via transnitrosylation from GAPDH-SNO translocated to the nucleus [75]. Nitrosylation of nuclear SIRT1 inhibited deacetylation of PGC1*α* in HEK293 cells. Mutational analysis on transfected cells with mouse SIRT1 plasmids identified C387 and C390 from the zinc tetrathiolate motif as the sites of S-nitrosylation. Surprisingly, C363 and C366 that also participate in zinc coordination were not susceptible to transnitrosylation. More recently, C371 and C374 from hSIRT1 (corresponding to C363 and C366 in mSIRT1) have been identified as the cysteines reduced by APE/Ref-1 to stimulate endothelial SIRT1 activity (although the other two cysteines involved in zinc ion coordination were not tested) [54].

When HepG2 cells transiently transfected with mouse SIRT1 WT were treated with increasing concentrations of CysNO or H_2_O_2_, decrease in p53 deacetylase activity was observed [76]. However, when cells were transfected with mSIRT1 mutants C61S, C318S, and/or C613S, the deacetylase activity was initially higher than with WT overexpression and less susceptible to oxidants [76]. The authors suggested reversible oxidative modification of SIRT1 forming GSH-adducts with these cysteine residues that are reverted by glutaredoxin 1. In this case, the reported cysteine residues oxidatively modified are not part of the Zn-binding motif.

Treatment of human epithelial cells with alkylating agent NEM diminished SIRT1 protein levels and free cysteine residues on immunoprecipitated SIRT1, although the specific residues modified were not identified [53].

Increased protein carbonylation of SIRT3 was found in liver mitochondrial extracts of ethanol-consuming mice [77]. The authors identified* in vitro* covalent modification of rSIRT3 by the electrophilic compound 4-hydroxynonenal at C280 (critical zinc-binding cysteine residue), resulting in inhibition of rSIRT3 activity [77].

More recently, mapping protein S-sulfenylation in cells treated with exogenous H_2_O_2_ as well as endogenous H_2_O_2_ (EGF treatment in A431 cells), SIRT6 was found among the most highly and consistently S-sulfenylated proteins [78]. Cysteine C18, a highly conserved residue close to the amino terminus, was identified as Cys-SOH that could form a covalent complex with HIF1*α* via disulfide bond, suggesting SIRT6-mediated redox control of HIF1*α* transcriptional activity [78].

Even though sirtuins do not have critical cysteine residues that participate in the mechanism of catalysis, modification of cysteine residues affects their activity, because it alters either the enzyme structure or the interaction with other proteins. The four cysteines in the zinc tetrathiolate motif, highly conserved, are essential for having a properly folded enzyme; thus, mutation of these cysteines to serine, not surprisingly, diminished deacetylase activity [54].

Another PTM (tyrosine nitration) on SIRT6 was recently reported [79]. Treatment of recombinant SIRT6 with the peroxynitrite donor SIN-1 revealed nitration of the enzyme and diminished activity. The authors identified tyrosine Y257 as one of the amino acid residues modified and mutation Y257F causes loss of deacetylase activity and susceptibility to nitration by SIN-1. Nitrated SIRT6 was also found in retina in a model of endotoxin-induced retinal inflammation [79].

### 4.3. Regulation of Sirtuins by Protein-Protein Interaction during Oxidative Stress

Oxidative stress regulates the activity of different sirtuins by altering their binding to regulatory proteins. From all sirtuins, the most extensively studied in terms of regulation by protein-protein interaction is SIRT1. The main protein regulators of SIRT1 described so far are DBC1 (deleted in breast cancer 1) [80] and AROS (active regulator of SIRT1) [81], and both have been involved in SIRT1-mediated response to oxidative stress [81, 82]. In the case of AROS, it was shown that its knock-down decreases SIRT1-mediated response to oxidative stress in cells, although it is not clear whether the protein plays an active role in such response or it is binding to SIRT1 the critical event. Oxidative stress also alters the interaction of SIRT1 with its protein inhibitor DBC1. Oxidative stress promotes phosphorylation of DBC1 (Thr454) by an ATM/ATR-dependent mechanism, increasing its affinity for SIRT1 and leading to sirtuin inhibition [82]. Interestingly, in mice, both obesity and aging [83, 84] promote SIRT1 binding to DBC1 [80], leading to a decrease in SIRT1 activity. Finally, it was shown recently that during oxidative stress SIRT1 can be inactivated by cytoplasmic sequestration and localization into caveolae by direct binding to caveolin-1 [85].

Thus, many different mechanisms might be operating to regulate SIRT1 activity during oxidative stress.

### 4.4. Alterations of Intracellular NAD Levels and Sirtuin Regulation during Oxidative Stress

NAD availability is key in the regulation of all sirtuins [86]. In fact, it has been shown that NAD levels decline during aging, obesity, and other metabolic diseases [87], affecting the activities of sirtuins in different tissues. Importantly, interventions that prevent NAD decline in tissues protect against metabolic and age-related diseases [87–91]. Genetic deletion [90] and also pharmacological inhibition of the protein CD38 [92], the main NAD glycohydrolase in mammalian tissues [92], activate SIRT1 [93] and protect against obesity and metabolic syndrome [90]. Similar results were found by inhibition of other major NAD-consuming enzymes in tissues like PARP-1 [91]. In fact, SIRT1 and PARP-1 activities can influence each other, since it has also been reported that SIRT1 can deacetylate PARP-1, decreasing its activity [94]. Furthermore, pharmacological treatment with NAD precursors, like nicotinamide mononucleotide (NMN) or nicotinamide riboside (NR), prevents NAD decline and protects against many aspects of metabolic syndrome, including glucose intolerance [87, 88]. Altogether, these results open the possibility of using NAD therapy for the treatment of metabolic and age-related diseases.

It is well established that aging and also metabolic disorders like obesity lead to an increased oxidative stress in tissues. In addition, it has been shown that NAD decline inversely correlates with oxidative stress during aging [95] and that oxidative stress negatively impacts on mitochondria, leading to NAD depletion in the matrix [96]. Interestingly, caloric restriction, an intervention shown to increase healthspan and to prevent metabolic syndrome, decreases oxidative stress leading to increased NAD levels and improving mitochondrial function by SIRT3-mediated increase in SOD2 activity [48].

## 5. Sirtuins as Pharmacological Targets for Metabolic and Age-Related Diseases

There has been considerable debate about pharmacological sirtuin activation and its effect on metabolism, cancer, and aging. The original observation that the polyphenol resveratrol and other small molecules (STACs, for sirtuin activating compounds) extend lifespan in* S. cerevisiae* through activation of Sir2 and that resveratrol could also activate human SIRT1 [97] puts sirtuins on the spot as ideal pharmacological targets for the treatment of aging and age-related diseases. Early on, an intense debate started about the role of resveratrol and other STACs as direct SIRT1 activators, since such activation appeared to rely on a specific activity assay and could not be reproduced by other means* in vitro* [98]. Since then, many molecular mechanisms have been proposed for SIRT1 activation by resveratrol* in vivo*, including direct SIRT1 activation [97, 99], activation of the AMPK-SIRT1 axis with NAD levels linking AMPK activation to SIRT1 activation [100], activation of the AMPK-SIRT1 axis through SIRT1 phosphorylation and dissociation from DBC1 [61], and SIRT1 activation through increase in cAMP levels by phosphodiesterase inhibition [101].

The development of novel, structurally different STACs by Sirtris Pharmaceuticals showed that SIRT1 activation by these new molecules (SRT1720, SRT1460, and SRT2183) prevents metabolic diseases in mice [99], and in the case of SRT1720, it was later shown that it also increases healthspan and lifespan in mice [102, 103]. Interestingly, the debate rose again about the specificity of these STACs for SIRT1 [104]. Recent research, however, has provided new evidence showing that these STACs, and even more potent new generations (STAC-5, STAC-9, and STAC-10), are indeed SIRT1 activators [105, 106].

Although the mechanism of action of resveratrol and other STACs may still need to be further investigated, it is clear that they provide beneficial effects against age-related disease* in vivo*. Resveratrol protects against high-fat diet induced obesity, type II diabetes, cardiovascular diseases, and cancer [99, 101, 107–114]. Similar results have been found with newly developed STACS [107, 107, 115, 116]. Interestingly, both resveratrol and the newly developed STACs decrease oxidative stress* in vitro* and* in vivo*, either by promoting antioxidant defenses or by improving mitochondrial function [103, 117–122].

The effect of STACS on human subjects has also been debated. Most of the evidence relies on studies conducted on volunteers who received resveratrol at different doses and for different periods of time. The evidence, reviewed in [153], shows that resveratrol might have some beneficial effects in humans, although its bioavailability is poor. Recently, phase I and II clinical trials were published with a new STAC (SRT2104), showing that it is well tolerated by the elderly, who showed decrease in cholesterol, LDL, and triglycerides levels, opening the possibility that STACs might become an available treatment for age-related diseases in humans [154, 155].

Finally, it is worth mentioning that SIRT6 might also be a pharmacological target for the treatment of age-related diseases, including inflammation, genomic stability, and cancer. The fact that SIRT6 is activated by fatty acids [2] might provide new avenues into the treatment of age-related diseases [156, 157].

## 6. Conclusions and Perspectives

Sirtuins are NAD-dependent deacylases that catalyze not only deacetylation of histones but also deacylation of other proteins including transcription factors and metabolic enzymes thereby regulating cell cycle, differentiation, metabolism, stress resistance, senescence, and aging. Fine regulation of expression and activity of sirtuins is critical to maintain cellular homeostasis. Although it is clear that sirtuins are modulated by oxidative stress, the molecular mechanisms are not well understood. Active sirtuins protect cells from ROS-induced damage via their product OAADPR/ADPR that inhibits mitochondrial ROS production and increases NADPH levels from pentose phosphate pathway. Mild oxidative stress induces sirtuin expression as a compensatory mechanism, while harsh or prolonged oxidant conditions result in dysfunctional modified sirtuins more prone to degradation by the proteasome. The increase in the NAD/NADH ratio under oxidative stress conditions can result in higher availability of the NAD cofactor, thus an apparent increase in sirtuin activity. Oxidative PTM of sirtuins have been identified, both* in vitro* and* in vivo*, to inhibit deacylase activity, although they can also affect the interaction with modulators, like SIRT1 with its endogenous inhibitor DBC1, resulting in a net increase of SIRT1 activity. Further research is needed to establish the mechanisms of redox regulation of sirtuins. Particularly interesting is to investigate redox modulation of SIRT3 in the mitochondrial matrix where most of cellular oxidants are formed. The fact that sirtuins can be activated, either by modulating NAD bioavailability in tissues or by pharmacological activation by small molecules, gives a therapeutic opportunity for the treatment of metabolic and age-related diseases.

## Figures and Tables

**Table 1:** General characteristics of mammalian sirtuins.

Human isoforms	Localization	Length	Conserved catalytic core position	Catalytic activity	Substrate	Function	References
SIRT1	Nucleus cytoplasm	747 aa	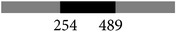	Deacetylase	p53, H3K9, H1K26, H4K16, PGC1*α*, SREBP-1c, PPAR*γ* NF-*κ*B, AKT, FOXO, HIF-1*α*, TFAM, AceCS1, APE1, and PARP-1	Lipogenesis ↓ Gluconeogenesis ↑↓ Lipolysis ↑ Inflammation ↓	[31, 42, 45, 94, 123–135]
SIRT2	Cytoplasm	352 aa	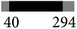	Deacetylase demyristoylase	PEPCK, *α*-tubulin, H4K16, and FOXO3a	Gluconeogenesis ↑ Control of mitotic exit	[37, 136–139]
SIRT3	Mitochondria	399 aa	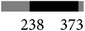	Deacetylase	LCAD, HMGCS2, SOD2, IDH2, PDC, and AceCS2	Lipid accumulation ↓ Fatty acid oxidation ↑ Ketone body production ↑ Oxidative stress ↓	[48, 132, 140–143]
SIRT4	Mitochondria	314 aa	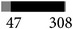	ADP-ribosyltransferase Lipoamidase	GDH, PDC	Insulin secretion ↓	[17, 144]
SIRT5	Mitochondria	310 aa	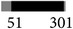	Desuccinylase Demalonylase Deglutarylase	CPS1	Urea cycle ↑	[18, 145, 146]
SIRT6	Nucleus endoplasmic reticulum	355 aa	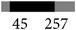	Depalmitoylase ADP-ribosyltransferase Deacetylase Demyristoylase	H3K9, H3K56, TNF*α*, and PARP-1	Glucose uptake ↓ Inflammation ↑ DNA reparation ↑	[16, 19, 147–150]
SIRT7	Nucleolus	400 aa	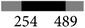	ADP-ribosyltransferase Deacetylase	PAF53, H3K18	RNA polymerase I transcription ↑	[151, 152]

SREBP-1c: sterol regulatory element binding protein c, PGC1*α*: peroxisome proliferator-activated receptor gamma coactivator 1 alpha, FOXO1: Forkhead box protein O1, PPAR*α*: peroxisome proliferator-activated receptor alpha, NF-*κ*B: nuclear factor kappa-light-chain-enhancer of activated B cells, AKT: protein kinase B, UCP-2: uncoupling protein 2, HIF-1*α*: hypoxia-inducible factor 1 alpha, PPAR*γ*: peroxisome proliferator-activated receptor gamma, TFAM: transcription factor A, mitochondrial, APE1: apurinic/apyrimidinic endonuclease 1, PARP-1: poly(ADP-ribose) polymerase 1, PEPCK: phosphoenolpyruvate carboxykinase, LCAD: long-chain acyl-CoA dehydrogenase, HMGCS2: 3-hydroxy-3-methylglutaryl-CoA synthase 2, SOD2: superoxide dismutase, IDH2: isocitrate dehydrogenase 2, PDC: pyruvate dehydrogenase complex, GDH: glutamate dehydrogenase, CPS1: carbamoyl-phosphate synthase 1, TNF*α*: tumor necrotic factor alpha, PAF53: RNA polymerase associated factor, and AceCS: acetyl-CoA synthetase.
